# Preparation of Eu_0.075_Tb_0.925_-Metal Organic Framework as a Fluorescent Probe and Application in the Detection of Fe^3+^ and Cr_2_O_7_^2−^

**DOI:** 10.3390/s21217355

**Published:** 2021-11-05

**Authors:** Jie Yin, Hongtao Chu, Shili Qin, Haiyan Qi, Minggang Hu

**Affiliations:** College of Chemistry and Chemical Engineering, Qiqihaer University, Qiqihaer 161006, China; yinjie199707@163.com (J.Y.); qinshili1103@163.com (S.Q.); lange19791@163.com (H.Q.); hmgxs@163.com (M.H.)

**Keywords:** Ln-MOFs, luminous sensing, Fe^3+^, Cr_2_O_7_^2−^

## Abstract

Luminescent Ln-MOFs (Eu_0.075_Tb_0.925_-MOF) were successfully synthesised through the solvothermal reaction of Tb(NO_3_)_3_·6H_2_O, Eu(NO_3_)_3_·6H_2_O, and the ligand pyromellitic acid. The product was characterised by X-ray diffraction (XRD), TG analysis, EM, X-ray photoelectron spectroscopy (XPS), and luminescence properties, and results show that the synthesised material Eu_0.075_Tb_0.925_-MOF has a selective ratio-based fluorescence response to Fe^3+^ or Cr_2_O_7_^2−^. On the basis of the internal filtering effect, the fluorescence detection experiment shows that as the concentration of Fe^3+^ or Cr_2_O_7_^2−^ increases, the intensity of the characteristic emission peak at 544 nm of Tb^3+^ decreases, and the intensity of the characteristic emission peak at 653 nm of Eu^3+^ increases in Eu_0.075_Tb_0.925_-MOF. The fluorescence intensity ratio (I_653_/I_544_) has a good linear relationship with the target concentration. The detection linear range for Fe^3+^ or Cr_2_O_7_^2−^ is 10–100 μM/L, and the detection limits are 2.71 × 10^−7^ and 8.72 × 10^−7^ M, respectively. Compared with the sensor material with a single fluorescence emission, the synthesised material has a higher anti-interference ability. The synthesised Eu_0.075_Tb_0.925_-MOF can be used as a highly selective and recyclable sensing material for Fe^3+^ or Cr_2_O_7_^2−^. This material should be an excellent candidate for multifunctional sensors.

## 1. Introduction

Heavy metals and inorganic anion pollutants in water pose hidden dangers to human health [1]. The United Nations Sustainable Development Goals set in September 2015 indicated that countries are expected to greatly improve human water quality by 2030. Thus, the detection of pollutants in water has become increasingly important. Fe^3+^ is one of the basic trace elements in humans. The lack or excess of this element can cause many physiological disorders, such as nausea, abdominal pain, anaemia, liver cirrhosis, and organ failure [2,3,4]. Salonen et al. [2] confirmed that elevated iron content is an important risk factor for acute myocardial infarction, Bijeh et al. [3] confirmed that the increased risk of cardiovascular disease is related to elevated iron content, and Jehn et al. [4] confirmed that elevated iron could lead to abnormal baseline metabolism. In addition, Cr_2_O_7_^2−^ is an important oxidant in laboratories and industry [5], and it is highly carcinogenic in the environment and harmful to the ecology, environment, and biological system [6,7,8,9]. Mansi et al. [6] confirmed that it is the second most abundant inorganic groundwater pollutant due to its wide application in many industrial fields, such as electroplating chrome, dyes, and leather tanning. Costa [7] confirmed that it is mutagenic and carcinogenic to organisms’ sexual function. Therefore, the selective sensing of Fe^3+^ and Cr_2_O_7_^2−^ in water quality has attracted growing attention from scholars. Many methods are used for the determination of Fe^3+^ and Cr_2_O_7_^2−^, such as atomic emission spectrometry, atomic absorption spectrometry, inductively coupled plasma mass spectrometry, electrochemical methods, and ion chromatography. However, these methods are complicated to operate, costly, and have a long detection time. Therefore, developing a simple and efficient method to determine Fe^3+^ and Cr_2_O_7_^2−^ is of practical significance. Fluorescence sensing technology can meet the requirements of new analysis and detection technology due to its high sensitivity, fast analysis speed, strong selectivity, simple operation, and low experimental cost. In recent years, it has received extensive attention [10].

Ln-MOFs materials refer to the self-assembly connection of metal ions and organic ligands by coordination bonds to form network complexes. Ln-MOFs materials have outstanding luminescence characteristics; that is, they have the advantages of large Stokes shift, high quantum yield and luminescence intensity, narrow emission spectrum range, flexible coordination mode, and long luminescence life. MOFs fluorescent probes are commonly used as sensors [11,12,13,14,15,16,17,18,19,20,21,22,23,24,25,26,27]. Hna et al. [26] synthesised Ce-MOF to detect Fe^3+^, and Gai et al. [27] synthesised dual-sensor Eu-MOF to detect Fe^3+^ and Cr_2_O_7_^2−^. The ratio fluorescent probe is based on measuring the ratio of the fluorescence intensity of two independent fluorescence emission peaks for quantitative analysis, which can effectively reduce the influence of excitation light, environment, and probe concentration changes, and greatly improve the accuracy of the method. At present, the usual design method of the Ln-MOFs ratio probe is to select two kinds of Ln^3+^ to synthesise by different molar ratios [28,29,30] or to combine Ln-MOFs with one or two substances with different fluorescence emission wavelengths, including carbon dots (CDs), quantum dots, and fluorescent dyes [31,32,33,34,35]. Zhang et al. [30] used two different molar ratios of Tb and Eu as the metal centre. 2,2′-bipyridine-6,6′-dicarboxylate acid (H_2_bpdc) is a ligand to synthesise Eu_0.6059_Tb_0.3941_-ZMOF, which can realise the selective detection of haemolysed phosphate (lysophosphatidic acid or LPA) in human plasma. Xu et al. [31] reported that CDs with strong fluorescence activity and Eu^3+^ were encapsulated in MOF-253, and the dual-emission ratio probe Eu^3+^/CDs@MOF-253 was synthesised to detect Hg^2+^. Therefore, the development of ratio fluorescent probe Ln-MOFs to detect Fe^3+^ and Cr_2_O_7_^2−^ has great application prospects.

The selective fluorescence detection of Fe^3+^ and Cr_2_O_7_^2−^ using ratio fluorescent probe Ln-MOFs is rarely reported in the literature. In this paper, luminescent Eu_0.075_Tb_0.925_-MOF was successfully synthesised by the solvothermal reaction of Tb(NO_3_)_3_·6H_2_O, Eu(NO_3_)_3_·6H_2_O, and ligand pyromellitic acid. Eu_0.075_Tb_0.925_-MOF was comprehensively characterised by XRD, thermogravimetric analysis (TG), elemental analysis, Fourier transform infrared spectroscopy (FTIR), transmission electron microscope (TEM), scanning electron microscope (SEM), and XPS. Eu_0.075_Tb_0.925_-MOF has excellent stability in aqueous solution, and it can detect Fe^3+^ and Cr_2_O_7_^2−^ in aqueous solution by dual-emission ratio fluorescence sensing, which provides a new idea for the fluorescence detection of Fe^3+^ and Cr_2_O_7_^2−^.

## 2. Materials and Methods

Commercially available reagents and solvents were used. XRD characterisation was performed to determine the regular arrangement of atoms or ions in the Eu_0.075_Tb_0.925_-MOF, which is one of the commonly used methods to explore the structure of matter. An elemental analyser was used for elemental analysis. FTIR was used to scan and analyse the range of 4000–400 cm^−1^ to determine the functional groups and chemical bonds of Eu_0.075_Tb_0.925_-MOF. The thermal stability of Eu_0.075_Tb_0.925_-MOF was analysed by TG, which was performed under N_2_ protection. TEM and SEM were used to observe the specific morphology of Eu_0.075_Tb_0.925_-MOF. The FL/FS900 fluorescence spectrometer was used to record the steady-state luminescence performance of Eu_0.075_Tb_0.925_-MOF. XPS and UV spectrophotometers were used to investigate the reaction mechanism.

Synthesis of Eu_0.075_Tb_0.925_-MOF: Product preparation was the first step. Eu_0.075_Tb_0.925_-MOF with Tb and Eu were prepared as the metal centre, and pyromellitic acid was prepared as the organic ligand as follows: Dissolved Tb(NO_3_)_3_·6H_2_O + Eu(NO_3_)_3_·6H_2_O (0.2 mmol), pyromellitic acid (0.2 mmol), DMF (8 mL), distilled water (4 mL), and CH_3_CH_2_OH (4 mL) were transferred to an autoclave (volume: 25 mL). The product was then sealed and heated in a 120 °C vacuum drying oven for 48 h and gradually cooled to ambient temperature. After the autoclave was opened, the product was collected after centrifugation, washed thoroughly with DMF and ethanol, paralleled three times, and dried. Thus, the target product Eu_0.075_Tb_0.925_-MOF was obtained.

## 3. Results and Discussion

### 3.1. XRD Characterisation

**Figure 1:**
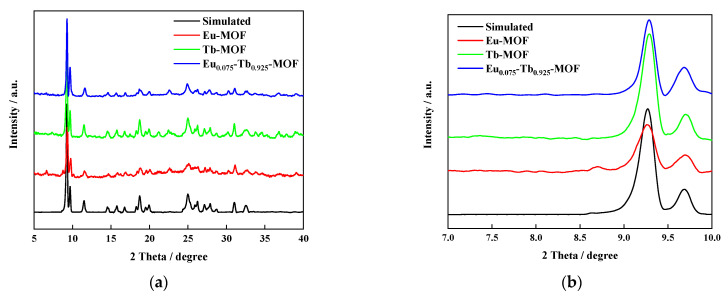
(**a**) XRD patterns of Ln-MOFs. (**b**) Enlarged version.

Eu-MOF, Tb-MOF, and Eu_0.075_Tb_0.925_-MOF combined with lanthanide nitrate and pyromellitic acid were prepared by the solvothermal method. Figure 1 shows the XRD patterns of Ln-MOFs. As shown in the figure, the 2θ diffraction angle peak positions of the simulated XRD pattern and the synthesised samples Eu-MOF, Tb-MOF, and Eu_0.075_Tb_0.925_-MOF are the same, and there are sharp peaks at the diffraction angles from 9 to 10. At the same time, the diffraction peaks 9 to 10 of the crystal synthesised by Silva et al. [36] are basically the same, indicating that the synthesised Eu_0.075_Tb_0.925_-MOF has high purity and good crystallinity [36,37,38,39].

### 3.2. TG Analysis

**Figure 2:**
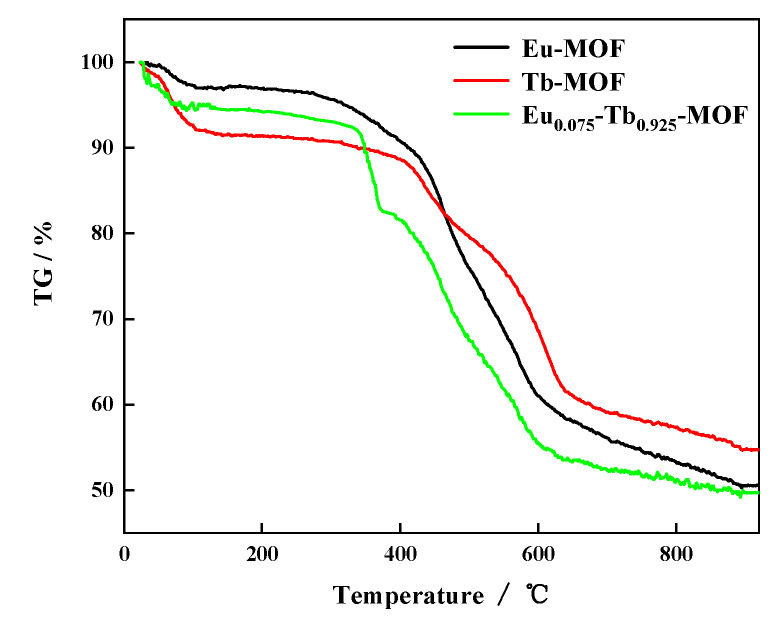
TG of Ln-MOFs.

Figure 2 shows the TG analysis results of Ln-MOFs. The weight loss of Ln-MOFs is mainly divided into two stages. Before 340 °C, Eu_0.075_Tb_0.925_-MOF has good thermal stability.

### 3.3. FTIR Analysis

**Figure 3:**
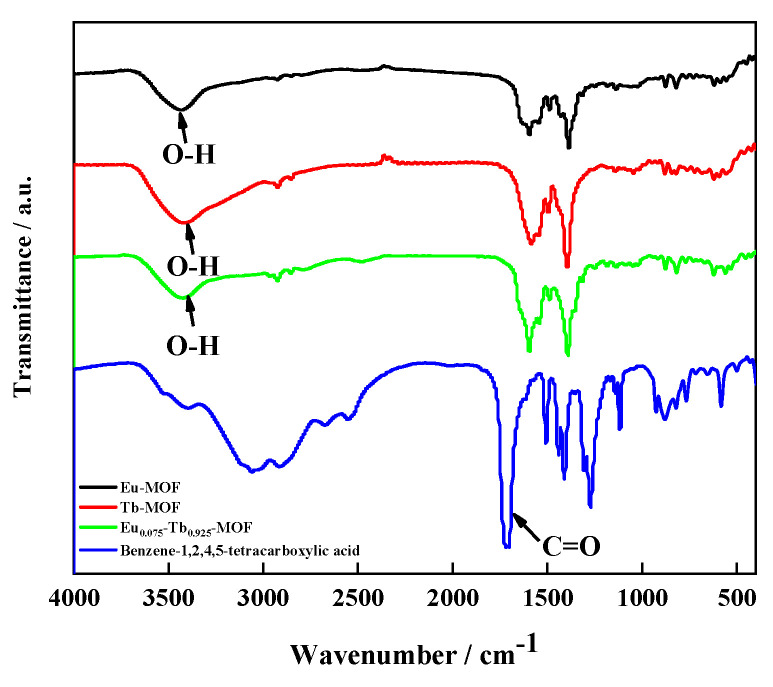
FTIR spectra of Benzene-1,2,4,5-tetracarboxylic acid and Ln-MOFs.

Figure 3 shows the FTIR spectrum of Ln-MOFs. Compared with the FTIR spectrum of pyromellitic acid, the main characteristic peaks in the FTIR spectrum of Eu_0.075_Tb_0.925_-MOF are similar to those of pyromellitic acid, but the C=O stretching vibration peak disappeared at 1720 cm^−1^ in the original pyromellitic acid spectrum (significantly weakened), thereby indicating that the carboxyl oxygen is coordinated with Tb and Eu atoms in the ligand.

### 3.4. Elemental Analysis and XPS

**Figure 4:**
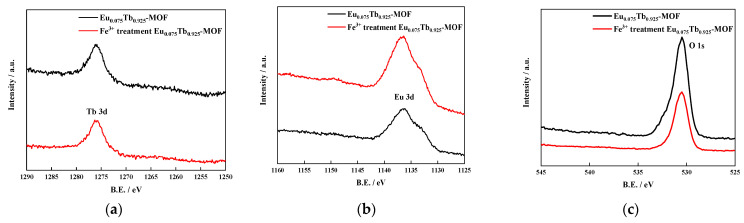
XPS of Eu_0.075_Tb_0.925_-MOF before and after Fe^3+^ addition: (**a**) Tb 3d, (**b**) Eu 3d, and (**c**) O 1s.

A comparison of elemental (Table 1) and XPS (Figure 4) analyses shows that, corresponding to the content of the element, the distribution ratio of Eu to Tb in Eu_0.075_Tb_0.925_-MOF is 0.075:0.925. The specific loadings of Tb(NO_3_)_3_ and Eu(NO_3_)_3_ are 42.74% and 3.46% respectively, and the cooling rate is 0.017 K/s.

### 3.5. EM Characterisation

**Figure 5:**
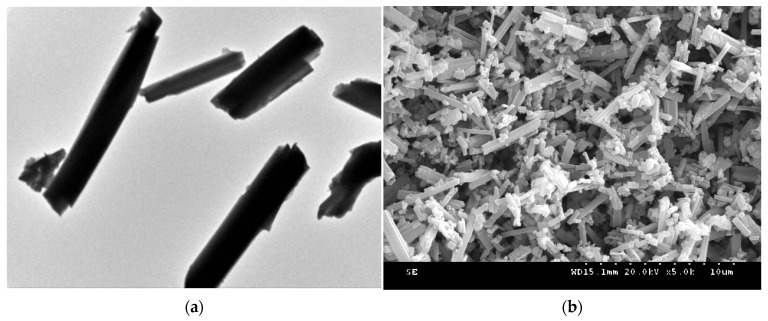
(**a**) TEM and (**b**) SEM of Eu_0.075_Tb_0.925_-MOF.

Figure 5 shows the TEM and SEM images of Eu_0.075_Tb_0.925_-MOF, which indicate that the prepared Eu_0.075_Tb_0.925_-MOF has a regular external morphology, a nanocolumn shape, and a diameter of about 500 nm.

### 3.6. Adsorption Characteristics of Eu_0.075_Tb_0.925_-MOF

**Figure 6:**
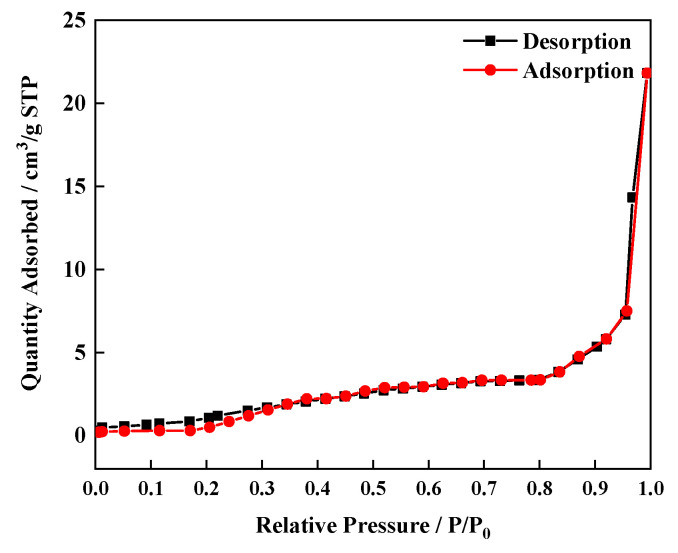
The N_2_ adsorption desorption isotherms of Eu_0.075_Tb_0.925_-MOF.

Figure 6 shows the N_2_ adsorption desorption isotherms of Eu_0.075_Tb_0.925_-MOF. The adsorption capacity increases slowly with the increase of pressure at the middle–high-pressure stage, indicating that Eu_0.075_Tb_0.925_-MOF is a porous material with an average pore size of 3.38 nm, a BJH average pore diameter of 20.99 nm, and a BET specific surface area of 12.9542 m^2^/g.

### 3.7. Photoluminescence Characteristics

**Figure 7:**
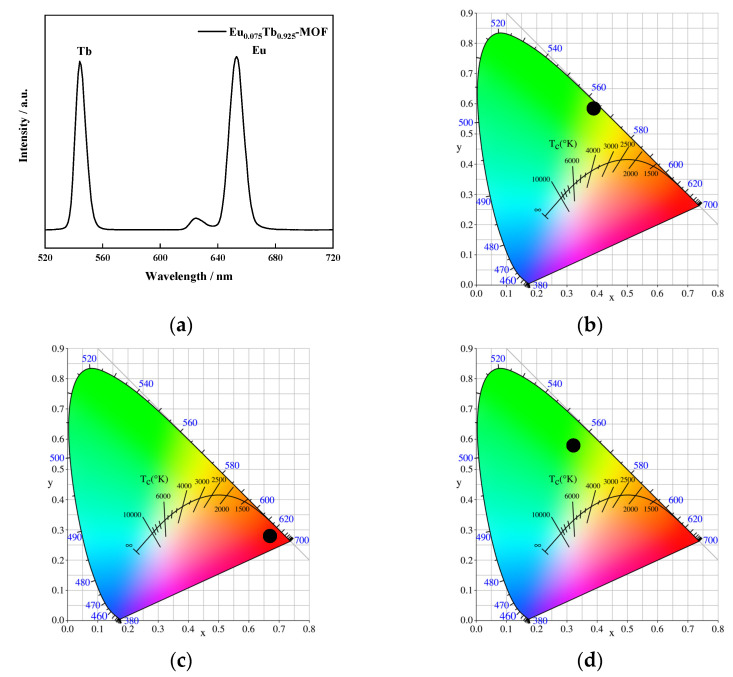
(**a**) Emission spectra of Eu_0.075_Tb_0.925_-MOF, (**b**) CIE of Eu_0.075_Tb_0.925_-MOF, (**c**) CIE of Eu-MOF, and (**d**) CIE of Tb-MOF.

Figure 7a shows the fluorescence emission spectrum of Eu_0.075_Tb_0.925_-MOF measured at ambient temperature. The figure shows that Eu_0.075_Tb_0.925_-MOF exhibits characteristic transitions of Tb^3+^ and Eu^3+^ under the excitation of 310 nm light, located at 544 and 653 nm respectively, showing the same intensity of fluorescence emission. This finding indicates that the ligand can effectively transfer energy to Tb^3+^ and Eu^3+^ at the same time [40,41,42,43,44].

As shown in the CIE diagram in Figure 7c,d, Eu-MOF shows red fluorescence, and Tb-MOF shows green fluorescence. When Eu^3+^ and Tb^3+^ synthesise Eu_0.075_Tb_0.925_-MOF at a ratio of 0.075:0.925, Eu_0.075_Tb_0.925_-MOF shows the intermediate colour of the two, which is a yellow-green fluorescence sensitive to the human eye. This material has potential application as a luminescent material and a light-sensitive material for naked-eye detection [45].

### 3.8. Fluorescence Sensing of Fe^3+^

**Figure 8:**
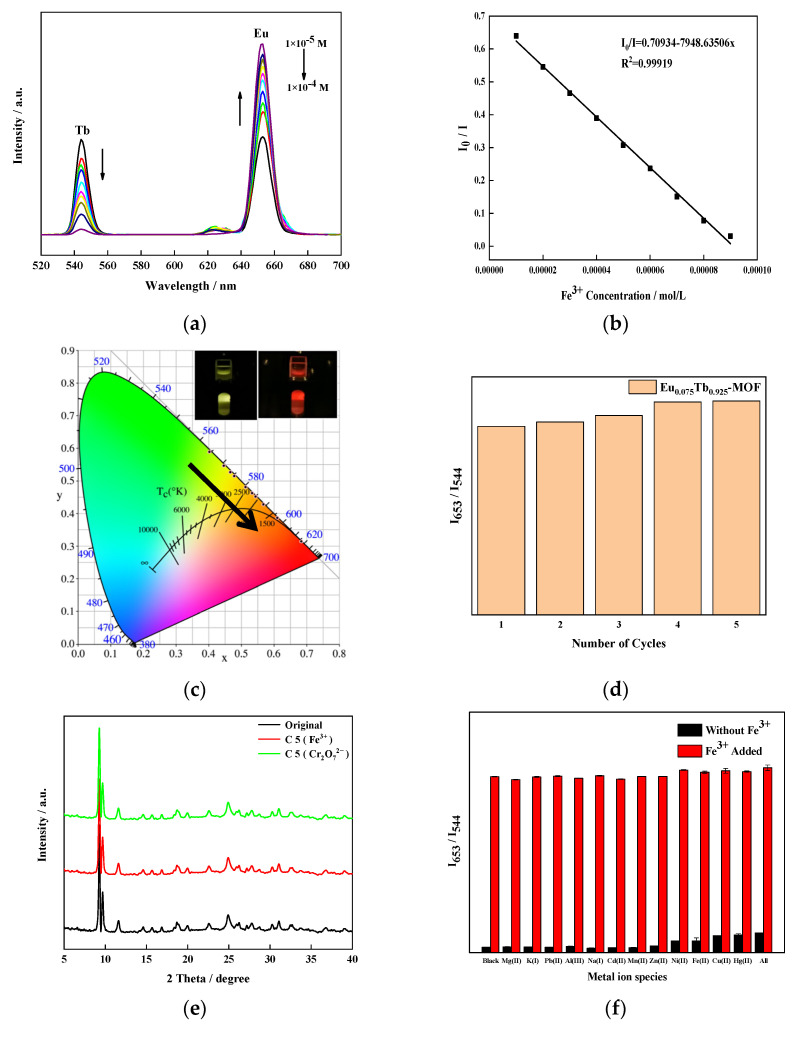
(**a**) The emission spectra of Eu_0.075_Tb_0.925_-MOF dispersions with different Fe^3+^ concentrations under 310 nm excitation light. (**b**) Calibration line with Fe^3+^(in the range of 10–100 μM/L), (**c**) CIE, (**d**) cycles of Eu_0.075_Tb_0.925_-MOF, (**e**) XRD pattern of Eu_0.075_Tb_0.925_-MOF after five cycles, and (**f**) I_Eu_/I_Tb_ histogram of Eu_0.075_Tb_0.925_-MOF dispersion containing metallic cations.

Gao and Ma [46,47] prepared Tb-MOF and used it for sensitive fluorescence sensing of Fe^3+^ and Cr_2_O_7_^2−^. On this basis, this paper designs a ratio fluorescent probe, Eu_0.075_Tb_0.925_-MOF, for the fluorescence sensing of Fe^3+^ and Cr_2_O_7_^2−^ to improve the measurement accuracy and expand the linear range of the test. To determine the fluorescence performance of Eu_0.075_Tb_0.925_-MOF to Fe^3+^, the fluorescence response of Eu_0.075_Tb_0.925_-MOF to Fe^3+^ was investigated, and the results are shown in Figure 8.

Figure 8a shows that with the increase of the Fe^3+^ concentration, the characteristic emission peak intensity of Tb^3+^ decreases at 544 nm, and the characteristic emission peak intensity of Eu^3+^ increases at 653 nm. The intensity at I_Eu_ = 653 nm and I_Tb_ = 544 nm is used to calculate the intensity change I_0_/I, where I_0_ (I_Eu0_/I_Tb0_) is the initial fluorescence intensity before fluorescence, and I (I_Eu_/I_Tb_) is the fluorescence intensity in the presence of Fe^3+^. Figure 8b shows that I_0_/I and Fe^3+^ present a linear relationship in the concentration range of 10–100 μM/L, and the linear regression equation is:

I_0_/I = 0.71 − 7948.64x. (1)

The limit of detection (LOD) of Fe^3+^ is evaluated by the equation 3S_b_/S, where S_b_ is the standard deviation of repeated detection of the original solution, and S is the slope of the linear fit. The LOD is calculated as 2.71 × 10^−7^ M. Figure 8c shows that the colour change trend of Eu_0.075_Tb_0.925_-MOF is yellow green–yellow–orange–red with the increase in Fe^3+^ concentration. This material is expected to achieve naked-eye detection of Fe^3+^.

The prepared Eu_0.075_Tb_0.925_-MOF was subjected to a cyclic application experiment, and KNO_3_ solution was used to wash the used materials. Figure 8d,e shows that the ratio of the luminous intensity of the material and the XRD did not change considerably, even after five cycles. Eu_0.075_Tb_0.925_-MOF is very stable in the sensing experiment.

The fluorescence sensing selectivity of Eu_0.075_Tb_0.925_-MOF to Fe^3+^ was investigated through the anti-interference experiment. The Eu_0.075_Tb_0.925_-MOF sample was immersed in NaX solution (Mg^2+^, K^+^, Pb^2+^, Al^3+^, Na^+^, Cd^2+^, Mn^2+^, Zn^2+^, Ni^2+^, Fe^2+^, Cu^2+^, Hg^2+^) at a concentration of 1 × 10^−4^ M. The results are shown in Figure 8f. Except for Fe^3+^, the luminous intensity ratio of Eu_0.075_Tb_0.925_-MOF exhibits almost no change after the addition of metal ions. However, when the same amount of Fe^3+^ was added to the Mg^2+^, K^+^, Pb^2+^, Al^3+^, Na^+^, Cd^2+^, Mn^2+^, Zn^2+^, Ni^2+^, Fe^2+^, Cu^2+^, and Hg^2+^ solution containing Eu_0.075_Tb_0.925_-MOF, the luminous intensity ratio of I_Eu_/I_Tb_ was significantly higher. This result shows that the sensing ability of Eu_0.075_Tb_0.925_-MOF on Fe^3+^ will not be interfered with by the presence of other metal ions. Therefore, Eu_0.075_Tb_0.925_-MOF has a high selectivity for Fe^3+^ in an aqueous solution.

### 3.9. Fluorescence Sensing of Cr_2_O_7_^2−^

**Figure 9:**
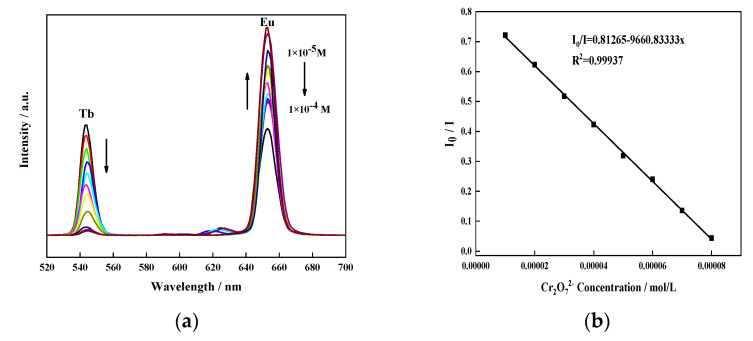

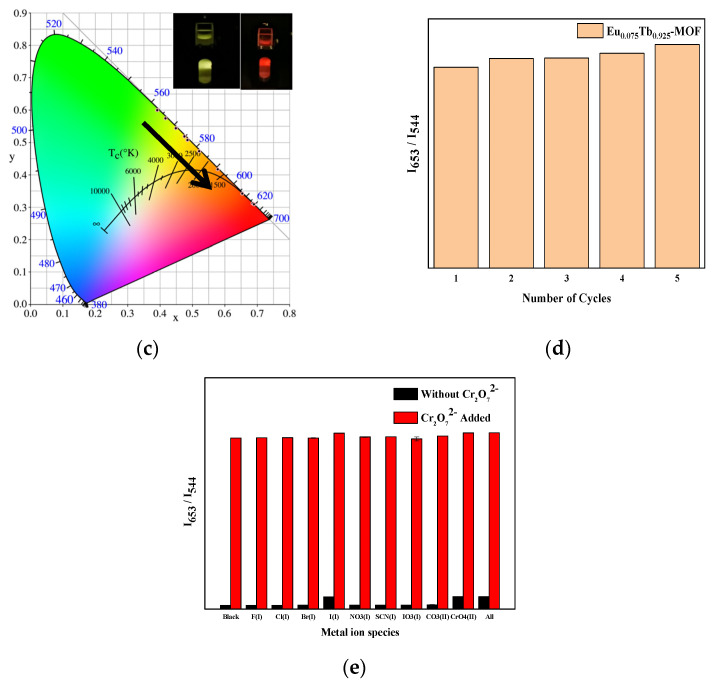
(**a**) The emission spectra of Eu_0.075_Tb_0.925_-MOF dispersions with different Cr_2_O_7_^2−^ concentrations under 310 nm excitation light. (**b**) Calibration line with Cr_2_O_7_^2−^(in the range of 10–100 μM/L), (**c**) CIE, (**d**) cycles of Eu_0.075_Tb_0.925_-MOF, and (**e**) I_Eu_/I_Tb_ histogram of Eu_0.075_Tb_0.925_-MOF dispersion containing anions.

To determine the fluorescence performance of Eu_0.075_Tb_0.925_-MOF to Cr_2_O_7_^2−^, the fluorescence response of Eu_0.075_Tb_0.925_-MOF to Cr_2_O_7_^2−^ was investigated, and the results are shown in Figure 9.

Figure 9a shows that with the increase in Cr_2_O_7_^2−^ concentration, the characteristic emission peak intensity of Tb^3+^ decreases at 544 nm, and the characteristic emission peak intensity of Eu^3+^ increases at 653 nm. At the same time, I_0_/I and Cr_2_O_7_^2−^ showed a linear correlation in the concentration range of 10–100 μM/L, the linear regression equation is:

I_0_/I = 0.81 − 9660.83x, (2)

and the LOD was 8.72 × 10^−7^ M. The CIE diagram in Figure 9c shows that with the increase in Cr_2_O_7_^2−^ concentration, the colour change trend of Eu_0.075_Tb_0.925_-MOF is yellow green–yellow–orange–red, which is expected to realise the naked-eye detection of Cr_2_O_7_^2−^.

A cyclic application experiment was performed on Eu_0.075_Tb_0.925_-MOF. Figure 9d shows that the luminous intensity ratio of Eu_0.075_Tb_0.925_-MOF does not change much after five cycles. Eu_0.075_Tb_0.925_-MOF was very stable in the sensing experiment.

Similarly, the fluorescence sensing selectivity of Eu_0.075_Tb_0.925_-MOF to Cr_2_O_7_^2−^ was investigated through the anti-interference experiment. Eu_0.075_Tb_0.925_-MOF was dispersed into a solution containing F^−^, Cl^−^, I^−^, Br^−^, NO_3_^−^, CrO_4_^2−^, SCN^−^, IO_3_^−^, CO_3_^2−^, and Cr_2_O_7_^2−^ with the same concentration. The results are shown in Figure 9e. Except for Cr_2_O_7_^2−^, the luminous intensity ratio of Eu_0.075_Tb_0.925_-MOF is almost unchanged after the addition of anions. However, when the same amount of Cr_2_O_7_^2−^ was added to the F^−^, Cl^−^, I^−^, Br^−^, NO_3_^−^, CrO_4_^2−^, SCN^−^, IO_3_^−^, and CO_3_^2−^ solution containing Eu_0.075_Tb_0.925_-MOF, the luminous intensity ratio of I_Eu_/I_Tb_ was significantly higher. This result shows that the sensing ability of Eu_0.075_Tb_0.925_-MOF on Cr_2_O_7_^2−^ will not be interfered with by the presence of other anions. Therefore, Eu_0.075_Tb_0.925_-MOF has a high selectivity for Cr_2_O_7_^2−^ in an aqueous solution.

### 3.10. Comparison with Other Sensors That Detect Fe^3+^ and Cr_2_O_7_^2−^ Ions

Compared with the Fe^3+^ and Cr_2_O_7_^2−^ detection methods used in other studies, as shown in Table 2, the prepared Eu_0.075_Tb_0.925_-MOF can reduce the effects of interference caused by excitation light, the environment, and probe concentration changes, and it has improved the detection accuracy relative to other methods.

### 3.11. Mechanism Study

**Figure 10:**
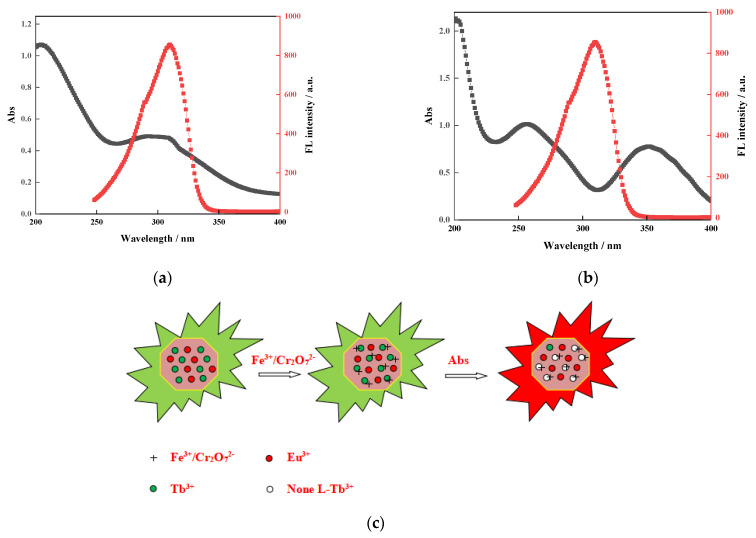
(**a**) Fluorescence excitation spectra of Eu_0.075_Tb_0.925_-MOF and UV-Vis absorption spectra of Fe^3+^. (**b**) Fluorescence excitation spectra of Eu_0.075_Tb_0.925_-MOF and UV-Vis absorption spectra of Cr_2_O_7_^2−^. (**c**) The mechanism of Fe^3+^ and Cr_2_O_7_^2−^ on Eu_0.075_Tb_0.925_-MOF fluorescence sensing.

The mechanism of Fe^3+^ and Cr_2_O_7_^2−^ on Eu_0.075_Tb_0.925_-MOF fluorescence sensing is examined. Figure 10a shows that the UV absorption spectrum of Fe^3+^ overlaps with the excitation spectrum of Eu_0.075_Tb_0.925_-MOF, which indicates that Fe^3+^ and Eu_0.075_Tb_0.925_-MOF are competitively adsorbed. At the same time, Figure 4 shows that Fe^3+^ is attached to the surface of Eu_0.075_Tb_0.925_-MOF and that the interaction between Fe^3+^ and the uncoordinated O atom in the ligand is weak. Eu_0.075_Tb_0.925_-MOF reduces the energy transfer from the ligand to Tb^3+^, and Tb^3+^ is quenched. As a result, the energy transfer from the ligand to Eu^3+^ is increased, and the characteristic red fluorescence of Eu^3+^ is displayed. Figure 10b shows that the UV absorption spectrum of Cr_2_O_7_^2−^ overlaps with the excitation spectrum of Eu_0.075_Tb_0.925_-MOF, which indicates that Cr_2_O_7_^2−^ and Eu_0.075_Tb_0.925_-MOF are competitively adsorbed. It will also cause the energy transfer from the ligand to Eu^3+^ to increase and show the characteristic red fluorescence of Eu^3+^.

### 3.12. Application in Actual Water Sample Analysis

The ratio fluorescent probe Eu_0.075_Tb_0.925_-MOF was used for Fe^3+^ and Cr_2_O_7_^2−^ in tap water. The results are shown in Table 3. The sample recovery rate is 101–114%, thereby showing that the established method has high accuracy and precision for the determination of Fe^3+^ and Cr_2_O_7_^2−^ content in actual samples.

## 4. Conclusions

The ratio fluorescent probe Eu_0.075_Tb_0.925_-MOF was synthesised in this experiment by using the solvothermal method and was used for Fe^3+^ and Cr_2_O_7_^2−^ determination. Mainly on the basis of the internal filtering effect, the characteristic fluorescence emission peak intensity of Tb^3+^ decreased, and the characteristic emission peak intensity of Eu^3+^ increased on Eu_0.075_Tb_0.925_-MOF as the concentration of Fe^3+^ and Cr_2_O_7_^2−^ increased. The ratio of the emission fluorescence intensity at the two wavelengths has a linear relationship with the target concentration, which realises the selective detection of Fe^3+^ and Cr_2_O_7_^2−^. The linear detection range was 10–100 μM, and the LOD was 2.71 × 10^−7^ and 8.72 × 10^−7^ M, respectively. The synthesised material was used as a ratio fluorescent probe, which can effectively eliminate background fluorescence interference in the detection process and improve the detection accuracy. The trend of the fluorescence colour change of the synthesised material during the detection process indicates that the material is expected to realise naked-eye detection of Fe^3+^ and Cr_2_O_7_^2−^.

## Figures and Tables

**Table 1:** Element analysis table.

Ln-MOFs	C	H	N	O	Eu/Tb
Eu-MOF	30.37%	1.87%	1.87%	26.64%	39.25%
Tb-MOF	22.01%	1.84%	1.13%	26.11%	48.91%
Eu_0.075_Tb_0.925_-MOF	23.53%	1.85%	1.56%	26.85%	46.21%

**Table 2:** Comparison of the reported methods for Fe^3+^ and Cr_2_O_7_^2−^ using Ln-MOFs.

Ln-MOFs	Detect Ion	LOD (M)	Ratio Fluorescent Probe	Linear Range	References
Eu_0.075_Tb_0.925_-MOF	Fe^3+^	2.71 × 10^−7^	Dual emission	10–100 μM (R^2^ = 0.99919, R^2^ = 0.99937)	This work
	Cr_2_O_7_^2−^	8.72 × 10^−7^			
Eu-MOF; Tb-MOF [Eu/Tb, 4,4′-(((5- carboxy-1,3-phenylene)bis(azanediyl))bis(carbonyl)) dibenzoic acid]	Fe^3+^	1 × 10^−5^	Single emission	0–1.0 mM (R^2^ = 0.9021, R^2^ = 0.9752)	[47]
	Cr_2_O_7_^2−^	8.94 × 10^−5^			
Eu-MOF [Eu, 5-(2′,5′-dicarboxylphenyl) picolinic acid ligand]	Fe^3+^	5.7 × 10^−7^	Single emission	0–50 μM (R^2^ = 0.9948, R^2^ = 0.9979)	[48]
	Cr_2_O_7_^2−^	4.2 × 10^−7^			
Tb-MOF [Tb,H3BTB]	Fe^3+^	1 × 10^−5^	Single emission	-	[49]
Eu-MOF [Eu, 2-aminoterephthalic acid 1,10-phenanthroline]	Fe^3+^	4.5 × 10^−5^	Single emission	0–0.25 mM (R^2^ = 0.992)	[50]
Tb-MOF [Tb, 2-(2-carboxyphenoxy)terephthalic acid]	Fe^3+^	2.0 × 10^−4^	Single emission	10^−4^–10^−3^ M (R^2^ = 0.978)	[51]
Eu-MOF [Eu, 2-(3′,4′-dicarboxylphenoxy)isophthalic acid, 4,4′-bis(imidazolyl) biphenyl	Fe^3+^	1.32 × 10^−5^	Single emission	0–10^−5^ M (R^2^ = 0.9885, R^2^ = 0.9927)	[52]
	Cr_2_O_7_^2−^	1.01 × 10^−5^			

**Table 3:** Determination of Fe^3+^ and Cr_2_O_7_^2−^ in real samples (*n* = 3).

Sample	Spiked (nM)	Found (nM)	Recovery (%)
Tap water (Fe^3+^)	20.0	22.1	110.5
	40.0	45.7	114.3
	60.0	61.6	102.7
	800	88.7	110.9
Tap water (Cr_2_O_7_^2−^)	20.0	20.9	104.5
	40.0	41.3	103.3
	60.0	60.9	101.5
	80.0	80.8	101.0
